# Supplementary material for: HSPS-10—Short Version of the Highly Sensitive Person Scale for Students Aged 12–25 Years
Source: Int J Environ Res Public Health. 2022 Nov 27;19(23):15775. doi: 10.3390/ijerph192315775 (PMC9739996; doi:10.3390/ijerph192315775)
Supplement: Supplementary file 1 [file ijerph-19-15775-s001.zip › ijerph-2045995-supplementary.pdf]

Supplementary Materials

Table S1: HSPS-10 QUESTIONS

Data badania ……………………

Kwestionariusz HSPS-10

SHORT POLISH VERSION OF THE HIGHLY SENSITIVE PERSON SCALE

KWESTIONARIUSZ OSOBA WYSOKO WRAŻLIWA – POLSKA SKRÓCONA WERSJA

(Autor: E.N. Aron; polska adaptacja: Monika Baryła-Matejczuk, Wiesław Poleszak, Robert Porzak)

……………………	……………………..
Wiek	Płeć

INSTRUKCJE: Odpowiedz na każde pytanie zgodnie z tym jak się czujesz, używając następującej skali:

**1**	**2**	**3**	**4**	**5**	**6**	**7**
**Zupełnie nie**			**Umiarkowanie**			**Zdecydowanie tak**

1.	Czy masz bogate, złożone życie wewnętrzne?	1	2	3	4	5	6	7
2.	Czy drażnią cię głośne dźwięki?	1	2	3	4	5	6	7
3.	Czy głęboko przeżywasz sztukę lub muzykę?	1	2	3	4	5	6	7
4.	Czy denerwujesz się kiedy musisz zrobić dużo rzeczy jednocześnie?	1	2	3	4	5	6	7
5.	Czy drażni Cię kiedy inni chcą od Ciebie zbyt wiele rzeczy na raz?	1	2	3	4	5	6	7
6.	Czy zmiany w Twoim życiu dezorganizują Cię?	1	2	3	4	5	6	7
7.	Czy zwracasz uwagę i cieszysz się z delikatnych lub pięknych zapachów, smaków, dźwięków lub dzieł sztuki?	1	2	3	4	5	6	7
8.	Czy źle się czujesz gdy trzeba robić wiele rzeczy jednocześnie?	1	2	3	4	5	6	7
9.	Czy przeszkadzają ci intensywne bodźce np. głośne dźwięki lub chaos?	1	2	3	4	5	6	7
10.	Czy stajesz się nerwowy i niepewny, a w efekcie osiągasz gorsze wyniki wtedy, gdy ktoś Ciebie obserwuje podczas rywalizacji lub wykonywania jakiegoś zadania?	1	2	3	4	5	6	7

Creative Commons Uznanie autorstwa CC BY, 2013
HSPS-10 Scale © 2020 M.Baryła-Matejczuk (For additional information email monika.baryla@wsei.lublin.pl)
